# Effects of Surgery on Prognosis of Young Women With Operable Breast Cancer in Different Marital Statuses: A Population-Based Cohort Study

**DOI:** 10.3389/fonc.2021.666316

**Published:** 2021-06-23

**Authors:** Junsheng Zhang, Ciqiu Yang, Yi Zhang, Fei Ji, Hongfei Gao, Xiaosheng Zhuang, Weiping Li, Weijun Pan, Bo Shen, Tingfeng Zhang, Yuanqi Chen, Kun Wang

**Affiliations:** ^1^ Department of Breast Cancer, Cancer Center, Guangdong Provincial People’s Hospital, Guangdong Academy of Medical Sciences, Guangzhou, China; ^2^ Shantou University Medical College, Shantou, China; ^3^ The Second School of Clinical Medicine, Southern Medical University, Guangzhou, China; ^4^ The First School of Clinical Medicine, Southern Medical University, Guangzhou, China

**Keywords:** breast cancer, breast-conserving surgery, mastectomy, breast reconstruction, survival

## Abstract

**Background:**

The influence of surgical approaches [including mastectomy, breast-conserving therapy (BCT) and post-mastectomy breast reconstruction (PMBR) on prognosis of young women (<40 years old) with operable breast cancer has not been determined yet, and this might vary in patients with different marital statuses. Therefore, we aimed to investigate the effect of surgery on survival outcomes for young women with operable breast cancer in different marital statuses.

**Methods:**

We used the Surveillance, Epidemiology, and End Results (SEER) database to identify young women with operable breast cancer between 2004 and 2016, who underwent mastectomy, BCT or PMBR. We assessed overall survival (OS) and breast cancer-specific survival (BCSS) using the Kaplan–Meier method and hazard ratios using multivariate Cox proportional hazard regression.

**Results:**

Compared to mastectomy, both of BCT and PMBR conferred better OS (BCT: HR = 0.79, 95%CI: 0.69–0.90, p <0.001; PMBR: HR = 0.70, 95%CI: 0.63–0.78, p <0.001) and BCSS (BCT: HR = 0.79, 95%CI: 0.69–0.91, p = 0.001; PMBR: HR = 0.73, 95%CI: 0.65–0.81, p <0.001), but there was no significant difference of survival between BCT and PMBR group. The survival benefit of BCT compared to mastectomy remained significant in unmarried young women (OS: HR = 0.68, 95%CI: 0.55–0.83, p <0.001; BCSS: HR = 0.69, 95%CI: 0.56–0.86, p = 0.001) but not in the married (OS: HR = 0.89, 95%CI: 0.75–1.05, p = 0.177; BCSS: HR = 0.89, 95%CI: 0.75–1.05, p = 0.161), while no matter married or not, PMBR group had better OS and BCSS than mastectomy group but not BCT group.

**Conclusion:**

Both of BCT and PMBR had improved survival compared to mastectomy for young women with operable breast cancer. The survival benefit of BCT compared to mastectomy remained significant in unmarried patients but not in married patients.

## Introduction

Nowadays, treatment strategies for breast cancer have been improved largely, including surgery, radiation, chemotherapy, endocrine therapy, target therapy and immune therapy (1). For operable breast cancer, surgical treatment, such as mastectomy alone, breast-conserving therapy (BCT) and post-mastectomy breast reconstruction (PMBR), is still considered to be the most significant treatment. Previous randomized controlled trials and large retrospective studies have demonstrated that BCT have equal or better survival outcomes compared with mastectomy (2–5), and there are also researches reported that PMBR brought survival benefits compared with mastectomy alone (6).

For breast cancer in young women, which are defined as women under the age of 40 at breast cancer diagnosis, the survival benefit of BCT compared with mastectomy was uncertain, though some studies had been reported that BCT brought better body image and less anxiety for young breast cancer survivors (7, 8). There were also few evidences regarding the survival outcomes of PMBR compared with mastectomy alone for young breast cancer patients. Therefore, the survival outcomes after different surgical options for young breast cancer patients need to be further investigated.

Psychosocial factors have been reported to be associated with survival outcomes of cancer patients, and marital status is one of the most important psychosocial factors for breast cancer patients (9). Previous studies have demonstrated that married patients had prolonged overall survival (OS) and breast cancer-specific survival (BCSS) compared with unmarried patients (including patients who were single, divorced, separated and widowed) (9, 10). Married patients could acquire more financial and emotional support and have better adherence when undergoing treatments (9), while unmarried patients, with less psychosocial support, might have higher expectations on treatments, especially when choosing surgical approaches. The body image after breast cancer local surgery seems to have more effects in young unmarried patients’ psychosocial life compared with those who are married, thus influencing their survival outcomes as well. Therefore, we hypothesized that the impact of surgical options on the prognosis of young breast cancer patients might be influenced by marital status.

The present study aimed to investigate the impact of surgical approaches (mastectomy, BCT or PMBR) on the overall survival (OS) and breast cancer-specific survival (BCSS) for young patients with operable breast cancer in different marital statuses using the Surveillance, Epidemiology, and End Results (SEER) database.

## Materials and Methods

### Study Population

**Figure 1:**
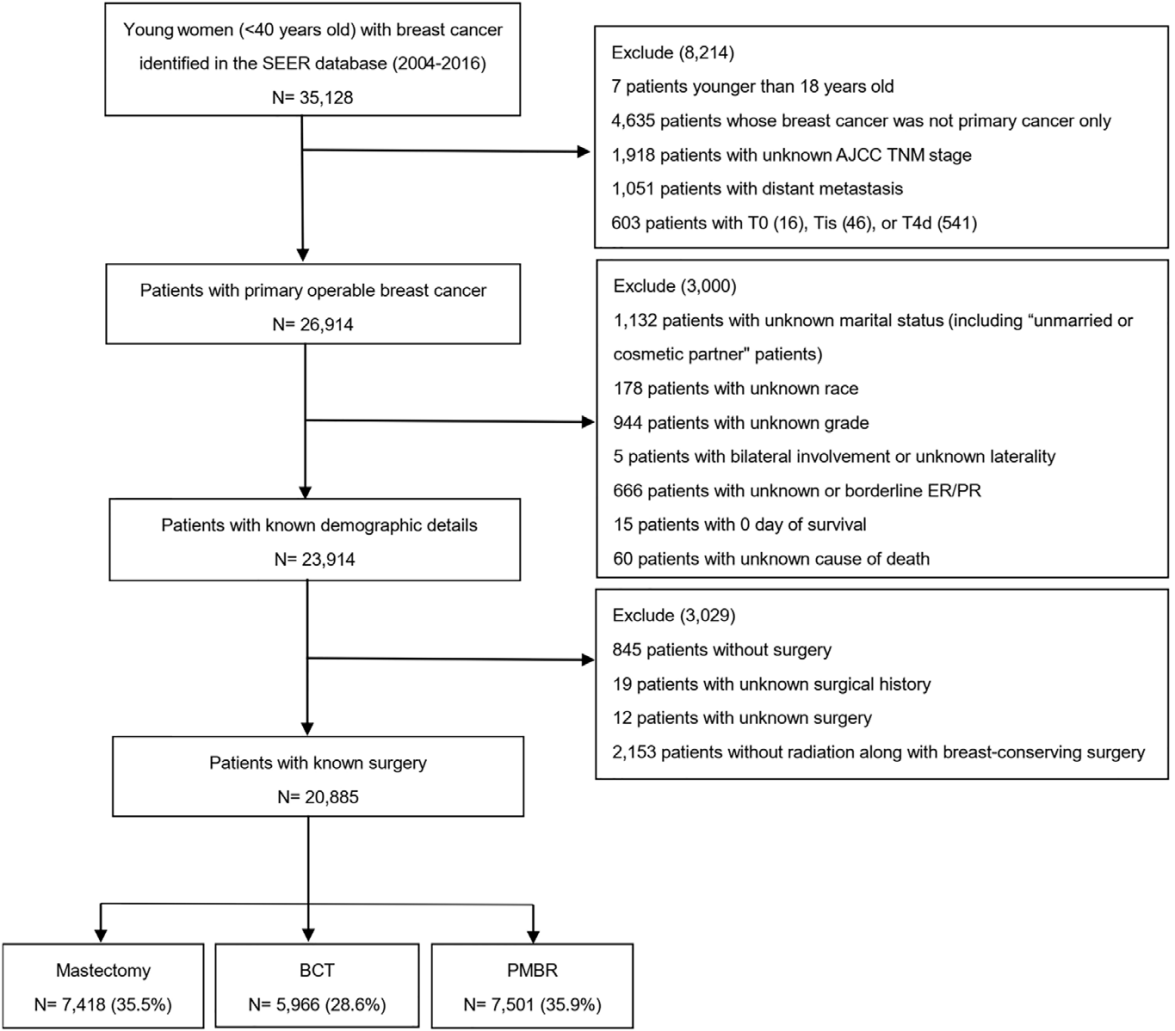
Flow diagram for creation of the study cohort. SEER, Surveillance, Epidemiology, and End Results; AJCC, American Joint Committee on Cancer; BCT, breast-conserving therapy; PMBR, post-mastectomy breast reconstruction.

We extracted data from the SEER database that was released in April 2019; specifically, the dataset named “Incidence-SEER 18 Res Research Data + Hurricane Katrina Impacted Louisiana Cases, Nov 2018 Sub (1975–2016 varying)” in the Case Listing and Frequency Sessions was obtained from the SEER*Stat software, version 8.3.6. The SEER database, including 18 tumor registries and representing approximately 28% of the population across the United States, contained information about patients’ demographics, characteristics of tumor, surgery type, hormone receptor status (HRs), survival months and vital status (11). Since the year 2004 was selected as the first year of the study given that several employed covariates were introduced in SEER in 2004, we identified 35,128 young women (<40 years old) diagnosed with breast cancer (International Classification of Diseases for Oncology, third edition (ICD-O-3) morphology code 8500, 8501, 8502, 8510, 8512, 8513, 8514, 8520, 8521, 8522, 8523, 8524, 8525, 8530, 8541 and 8543) from January 2004 to November 2016[9]. Then, only patients with primary operable breast cancer were included according to the 6th edition AJCC system for cases between 2004 and 2009, and the 7th edition for cases between 2010 and 2016. Patients with unknown details including marital status, race, tumor grade, HRs, surgery and cause of death and those without radiation along with breast-conserving surgery were excluded. Finally, 20,885 cases were selected into our study, and the entire cohort was divided into three groups according to their surgery type: mastectomy, BCT and PMBR (**Figure 1**). Marital status was categorized as either married or unmarried, and the unmarried included patients who were single, divorced, separated and widowed.

### Statistical Analysis

The patients’ baseline characteristics among mastectomy, BCT and PMBR group were compared using Pearson’s chi-square test. Survival outcomes, including OS and BCSS, were examined using the Kaplan–Meier method and compared among the three surgical groups using Log-rank tests. Meanwhile, the survival outcomes among the three surgical groups were further analyzed in subgroups stratified by marital status. Hazard ratios (HR) with 95% confidence intervals (CIs) to assess the survival difference among different surgical groups were calculated using multivariate Cox proportional hazard regression. A two-sided P value <0.05 was considered to indicate statistical significance. All analyses in our study were performed using Statistical Product and Service Solutions (SPSS) software (version 26.0).

## Results

### Patients’ Characteristics

Some 20,885 young women with primary operable breast cancer were included in our study, among which 7,418 (35.5%) underwent mastectomy, 5,966 (28.6%) underwent BCT and 7,501 (35.9%) underwent PMBR. The median follow-up time was 66 months. The patients’ characteristics including demographics, age of diagnosis, characteristics of tumor, surgery approach, radiation, and chemotherapy are showed in **Table 1**. Most patients were 30–39 years old (89.6%), married (64.2%), White people (73.1%), in AJCC stage II (49.9%) and had poorly differentiated or undifferentiated tumor (57.8%). Among the three surgical groups, BCT group had highest percentage of unmarried patients (39.5%), while mastectomy group had 35.8% and PMBR group had 32.9%. Consistent with the entire cohort, approximately half of the patients in each group were AJCC stage II; besides, the mastectomy group had more stage III patients (31.6%) while BCT group and PMBR group had more stage I patients (38.6 and 32.2%, respectively). The mastectomy group had a higher percentage of larger tumor size (>2 cm) (67.2%) than BCT group (48.1%) and PMBR group (54.9%).

**Table 1:** Comparison of baseline characteristics of operable breast cancer among various surgical groups.

Characteristics	Total (%)	MAS (%)	BCT (%)	PMBR (%)	p value
Overall	20,885	7,418	5,966	7,501	
Age (years)					<0.001
18–29	2,174 (10.4)	785 (10.6)	516 (8.6)	873 (11.6)	
30–39	18,711 (89.6)	7,418 (89.4)	5,450 (91.4)	6,628 (88.4)	
Marital status					<0.001
Married	13,402 (64.2)	4,761 (64.2)	3,607 (60.5)	5,034 (67.1)	
Unmarried	7,483 (35.8)	2,657 (35.8)	2,359 (39.5)	2,467 (32.9)	
Race					<0.001
White	15,272 (73.1)	5,316 (71.7)	4,169 (69.9)	5,787 (77.1)	
Black	2,941 (14.1)	1,068 (14.4)	959 (16.1)	914 (12.2)	
Others	2,672 (12.8)	1,034 (13.9)	838 (14.0)	800 (10.7)	
Year of Diagnosis					<0.001
2004–2009	8,971 (43.0)	3,684 (49.7)	3,098 (51.9)	2,189 (29.2)	
2010–2016	11,914 (57.0)	3,734 (50.3)	2,868 (48.1)	5,312 (70.8)	
Grade					<0.001
Well differentiated	1,560 (7.5)	464 (6.3)	564 (9.5)	532 (7.1)	
Moderately differentiated	7,245 (34.7)	2,410 (32.5)	2,001 (33.5)	2,834 (37.8)	
Poorly differentiated	11,886 (56.9)	4,450 (60.0)	3,346 (56.1)	4,090 (54.5)	
Undifferentiated/Anaplastic	194 (0.9)	94 (1.3)	55 (0.9)	45 (0.6)	
AJCC stage					<0.001
I	6,115 (29.3)	1,398 (18.8)	2,302 (38.6)	2,415 (32.2)	
II	10,426 (49.9)	3,673 (49.5)	3,068 (51.4)	3,685 (49.1)	
III	4,344 (20.8)	2,347 (31.6)	596 (10.0)	1,401 (18.7)	
Tumor size					<0.001
≤2 cm	8,822 (42.2)	2,390 (32.2)	3,089 (51.8)	3,343 (44.6)	
>2 cm,≤5 cm	9,456 (45.3)	3,614 (48.7)	2,631 (44.1)	3,211 (42.8)	
>5 cm	2,519 (12.1)	1,374 (18.5)	239 (4.0)	906 (12.1)	
Unknown	88 (0.4)	40 (0.5)	7 (0.1)	41 (0.5)	
LN status					<0.001
Negative	10,799 (51.7)	2,968 (40.0)	3,779 (63.3)	4,052 (54.0)	
Positive	9,773 (46.8)	4,339 (58.5)	2,099 (35.2)	3,335 (44.5)	
No examined/Unknown	313 (1.5)	111 (1.5)	88 (1.5)	114 (1.5)	
HRs					<0.001
ER+/PR+	12,362 (59.2)	4,141 (55.8)	3,592 (60.2)	4,629 (61.7)	
ER+/PR−	2,162 (10.4)	811 (10.9)	500 (8.4)	851 (11.3)	
ER−/PR+	407 (1.9)	150 (2.0)	112 (1.9)	145 (1.9)	
ER−/PR−	5,954 (28.5)	2,316 (31.2)	1,762 (29.5)	1,876 (25.0)	
Radiation					<0.001
No	9,210 (44.1)	4,197 (56.6)	0 (0.0)	5,013 (66.8)	
Yes	11,675 (55.9)	3,221 (43.4)	5,966 (100.0)	2,488 (33.2)	
Chemotherapy					<0.001
No/Unknown	3,771 (18.1)	1,158 (15.6)	1,119 (18.8)	1,494 (19.9)	
Yes	17,114 (81.9)	6,260 (84.4)	4,847 (81.2)	6,007 (80.1)	

MAS, mastectomy; BCT, breast-conserving therapy; PMBR, post-mastectomy breast reconstruction; AJCC, American Joint Committee on Cancer; LN, lymph node; HRs, hormone receptor status; ER, estrogen receptor; PR, progesterone receptor.

### Effects of Surgery on Survival Outcomes in Overall and Stratified by Marital Status

**Figure 2:**
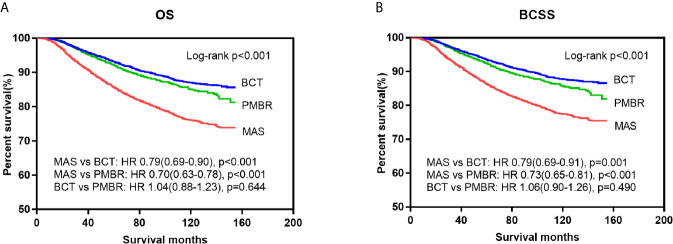
Kaplan–Meier survival curves: OS **(A)** and BCSS **(B)** of young women with operable breast cancer according to surgical type. OS, overall survival; BCSS, breast cancer-specific survival; MAS, mastectomy; BCT, breast-conserving therapy; PMBR, post-mastectomy breast reconstruction; HR, hazard ratios.

**Figure 3:**
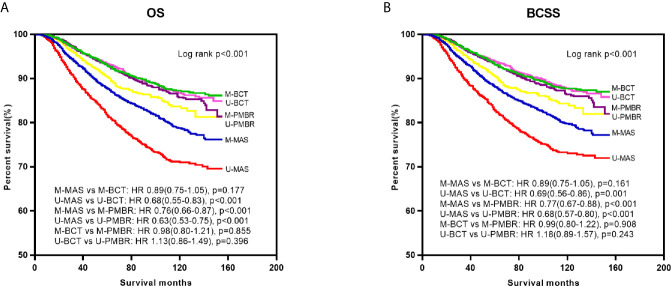
Kaplan–Meier survival curves: OS **(A)** and BCSS **(B)** of young women with operable breast cancer according to surgical type and marital status. OS, overall survival; BCSS, breast cancer-specific survival; M-MAS, married patients who underwent mastectomy; U-MAS, unmarried patients who underwent mastectomy; M-BCT, married patients who underwent breast-conserving therapy; U-BCT, unmarried patients who underwent breast-conserving therapy; M-PMBR, married patients who underwent post-mastectomy breast reconstruction; U-PMBR, unmarried patients who underwent post-mastectomy breast reconstruction; HR, hazard ratios.

Kaplan–Meier curves were generated by surgical approach to estimate OS and BCSS of patients with operable breast cancer. In log‐rank tests, the BCT and PMBR group showed significantly (P <0.001) better OS and BCSS than mastectomy group, while less significant difference of OS and BCSS was observed between the BCT and PMBR group (**Figure 2**). After adjusting the possible confounding variables via multivariate Cox regression analysis, it turned out that compared to mastectomy, both of BCT and PMBR conferred better OS (BCT: HR = 0.79, 95%CI:0.69–0.90, p <0.001; PMBR: HR = 0.70, 95%CI: 0.63<0.78, p <0.001) and BCSS (BCT: HR = 0.79, 95%CI: 0.69<0.91, p = 0.001; PMBR: HR = 0.73, 95%CI: 0.65–0.81, p <0.001), but there was no significant difference of survival between BCT group and PMBR group (OS: HR = 1.04, 95%CI: 0.88<1.23, p = 0.644; BCSS: HR = 1.06, 95%CI: 0.90–1.26, p = 0.490) (**Figure 2**). The superiority of BCT in survival outcomes compared to mastectomy remained significant in unmarried young women (OS: HR = 0.68, 95%CI: 0.55–0.83, p <0.001; BCSS: HR = 0.69, 95%CI: 0.56–0.86, p = 0.001) but not in the married (OS: HR = 0.89, 95%CI: 0.75–1.05, p = 0.177; BCSS: HR = 0.89, 95%CI: 0.75–1.05, p = 0.161), while no matter married or not, PMBR group had better OS and BCSS than mastectomy group but not BCT group (**Figure 3**).

### Effects of Surgery Stratified by Demographic and Pathological Subgroups

To further investigate the prognostic effect of surgery on survival by different demographic and pathological subgroups, we also stratified all cases according to age, race, AJCC stage, HRs, and whether receiving chemotherapy or not and conducted multivariate analyses (**Tables 2** and **3**). Compared with mastectomy, better OS of PMBR was observed in almost all subgroups except for HRs of ER+/PR− and non-chemotherapy group, while the superiority of BCT in OS was existed in all subgroups of age and race, and in stage I/II, HRs of ER+/PR+ and chemotherapy group. As for BCSS, the superiority of BCT was noticed in all age subgroups, in black people, HRs of ER+/PR+, and chemotherapy group compared with mastectomy; the benefit of PMBR in BCSS was significant in all age subgroups, in white and black people, stages I and III, all HRs except ER+/PR−, and chemotherapy group. In addition, there was no significant difference of OS and BCSS between BCT and PMBR in almost all subgroups except for stage I, in which PMBR had worse survival than BCT (OS: HR = 2.24, 95%CI: 1.12–4.47, p = 0.022; BCSS: HR = 2.51, 95%CI: 1.24–5.05, p = 0.010).

**Table 2:** Effects of surgery in overall survival by demographic and pathological subgroups.

OS	BCT *vs* MAS		PMBR *vs* MAS		PMBR *vs* BCT	
Variable	AHR* (95%CI)	p value	AHR* (95%CI)	p value	AHR* (95%CI)	p value
Age (years)						
18–29	0.66 (0.46–0.95)	0.024	0.67 (0.50–0.90)	0.008	0.90 (0.56–1.43)	0.650
30–39	0.81 (0.70–0.93)	0.003	0.70 (0.63–0.79)	<0.001	1.07 (0.89–1.28)	0.468
Race						
White	0.85 (0.72–0.99)	0.041	0.74 (0.65–0.83)	<0.001	1.02 (0.84–1.24)	0.846
Black	0.72 (0.54–0.94)	0.017	0.62 (0.49–0.79)	<0.001	1.12 (0.76–1.64)	0.561
Others	0.64 (0.43–0.96)	0.029	0.62 (0.42–0.92)	0.017	1.04 (0.58–1.85)	0.906
AJCC stage						
I	0.37 (0.21–0.65)	0.001	0.59 (0.41–0.84)	0.003	2.24 (1.12–4.47)	0.022
II	0.75 (0.62–0.91)	0.004	0.82 (0.70–0.96)	0.013	1.22 (0.96–1.55)	0.110
III	0.84 (0.69–1.02)	0.070	0.65 (0.56–0.76)	<0.001	0.85 (0.67–1.08)	0.187
HRs						
ER+/PR+	0.73 (0.60–0.89)	0.002	0.73 (0.62–0.86)	<0.001	1.09 (0.85–1.41)	0.497
ER+/PR−	0.75 (0.48–1.16)	0.197	0.85 (0.63–1.14)	0.273	1.45 (0.87–2.40)	0.151
ER−/PR+	0.74 (0.34–1.61)	0.440	0.27 (0.13–0.56)	<0.001	0.46 (0.14–1.57)	0.216
ER−/PR−	0.85 (0.70–1.03)	0.098	0.68 (0.58–0.81)	<0.001	0.92 (0.71–1.19)	0.505
Chemotherapy						
No/Unknown	0.97 (0.48–1.96)	0.931	0.74 (0.51–1.07)	0.106	1.74 (0.66–4.55)	0.260
Yes	0.78 (0.69–0.89)	<0.001	0.70 (0.63–0.79)	<0.001	1.02 (0.86–1.21)	0.816

*With adjustment for race, age, marital status, T stage, N stage, histological grade, hormone receptor status, tumor size, surgery, radiation and chemotherapy.

OS, overall survival; AHR, adjusted hazard ratios; CI, confidential interval; MAS, mastectomy; BCT, breast-conserving therapy; PMBR, post-mastectomy breast reconstruction; HRs, hormone receptor status; ER, estrogen receptor; PR, progesterone receptor.

**Table 3:** Effects of surgery in breast cancer-specific survival by demographic and pathological subgroups.

BCSS	BCT *vs* MAS		PMBR *vs* MAS		PMBR *vs* BCT	
Variable	AHR* (95%CI)	p value	AHR* (95%CI)	p value	AHR* (95%CI)	p value
Age (years)						
18–29	0.68 (0.47–0.98)	0.038	0.71 (0.53–0.96)	0.026	0.94 (0.58–1.52)	0.804
30–39	0.81 (0.70–0.94)	0.004	0.73 (0.65–0.82)	<0.001	1.09 (0.91–1.31)	0.356
Race						
White	0.86 (0.73–1.01)	0.074	0.76 (0.67–0.86)	<0.001	1.01 (0.83–1.24)	0.892
Black	0.69 (0.52–0.92)	0.011	0.64 (0.49–0.82)	<0.001	1.33 (0.91–1.96)	0.142
Others	0.65 (0.43–0.98)	0.040	0.68 (0.46–1.01)	0.053	1.15 (0.64–2.07)	0.640
AJCC stage						
I	0.34 (0.19–0.62)	<0.001	0.61 (0.42–0.88)	0.009	2.51 (1.24–5.05)	0.010
II	0.75 (0.62–0.92)	0.006	0.87 (0.74–1.03)	0.095	1.24 (0.97–1.59)	0.088
III	0.83 (0.68–1.01)	0.065	0.66 (0.56–0.77)	<0.001	0.87 (0.68–1.11)	0.257
HRs						
ER+/PR+	0.76 (0.62–0.93)	0.008	0.76 (0.64–0.89)	0.001	1.08 (0.83–1.40)	0.563
ER+/PR−	0.81 (0.51–1.27)	0.352	0.88 (0.65–1.19)	0.399	1.49 (0.89–2.49)	0.127
ER−/PR+	0.80 (0.35–1.79)	0.578	0.30 (0.15–0.62)	0.001	0.48 (0.14–1.69)	0.255
ER−/PR−	0.81 (0.67–0.99)	0.043	0.71 (0.60–0.84)	<0.001	0.96 (0.74–1.25)	0.784
Chemotherapy						
No/Unknown	0.82 (0.39–1.74)	0.604	0.73 (0.50–1.07)	0.108	2.04 (0.70–5.90)	0.189
Yes	0.79 (0.69–0.91)	0.001	0.73 (0.65–0.82)	<0.001	1.04 (0.88–1.24)	0.659

*With adjustment for race, age, marital status, T stage, N stage, histological grade, hormone receptor status, tumor size, surgery, radiation and chemotherapy.

OS, overall survival; AHR, adjusted hazard ratios; CI, confidential interval; MAS, mastectomy; BCT, breast-conserving therapy; PMBR, post-mastectomy breast reconstruction; HRs, hormone receptor status; ER, estrogen receptor; PR, progesterone receptor.

### Effect of Various Factors on Survival Outcomes

Univariate analysis and adjusted multivariate analysis showed that unmarried status, black people, higher tumor grade (poorly differentiated or undifferentiated), lager tumor size (>2 cm), AJCC stage III and positive lymph node were independent risk factors for OS and BCSS, while receiving BCT or PMBR was protective factor for OS and BCSS (**Table 4**). In univariate analysis, receiving radiation or chemotherapy were associated with lower OS and BCSS; however, after adjustment for confounding variables with multivariate analysis, receiving radiation was proved to have no significant effect in survival outcomes of young breast cancer patients while receiving chemotherapy had little effect in either OS or BCSS (HR = 1.18, 95%CI: 1.00–1.38, p = 0.049; HR = 1.18, 95%CI: 1.00–1.40, p = 0.047; respectively).

**Table 4:** Univariate and multivariate analysis of OS and BCSS for young women with operable breast cancer diagnosed between 2004 and 2016.

Characteristics	OS				BCSS			
	Univariate Analysis		Multivariate Analysis^*^		Univariate Analysis		Multivariate Analysis^*^	
	HR^†^ (95%CI)	p value	HR^†^ (95%CI)	p value	HR^†^ (95%CI)	p value	HR^†^ (95%CI)	p value
Age (years)								
18–29	Reference	–	Reference	–	Reference	–	Reference	–
30–39	0.78 (0.72–0.85)	<0.001	0.89 (0.78–1.01)	0.081	0.78 (0.71–0.85)	<0.001	0.89 (0.78–1.02)	0.081
Marital status								
Married	Reference	–	Reference	–	Reference	–	Reference	–
Unmarried	1.30 (1.19–1.41)	<0.001	1.22 (1.12–1.34)	<0.001	1.27 (1.17–1.39)	<0.001	1.20 (1.10–1.32)	<0.001
Race								
White	Reference	–	Reference	–	Reference	–	Reference	–
Black	1.67 (1.50–1.85)	<0.001	1.33 (1.19–1.49)	<0.001	1.63 (1.46–1.82)	<0.001	1.31 (1.17–1.47)	<0.001
Others	0.85 (0.73–0.98)	0.021	0.89 (0.77–1.02)	0.100	0.84 (0.73–0.98)	0.022	0.89 (0.76–1.03)	0.106
Year of Diagnosis								
2004–2009	Reference	–	Reference	–	Reference	–	Reference	–
2010–2016	0.97 (0.88–1.07)	0.549	1.06 (0.96–1.17)	0.256	0.96 (0.87–1.06)	0.426	1.05 (0.95–1.16)	0.379
Grade								
Well differentiated	Reference	–	Reference	–	Reference	–	Reference	–
Moderately differentiated	2.87 (2.09–3.95)	<0.001	1.93 (1.40–2.66)	<0.001	3.15 (2.24–4.44)	<0.001	2.09 (1.48–2.95)	<0.001
Poorly differentiated	5.34 (3.91–7.28)	<0.001	2.59 (1.88–3.55)	<0.001	5.92 (4.24–8.28)	<0.001	2.81 (1.99–3.95)	<0.001
Undifferentiated/Anaplastic	5.30 (3.40–8.26)	<0.001	2.55 (1.63–4.00)	<0.001	6.05 (3.80–9.63)	<0.001	2.85 (1.78–4.57)	<0.001
AJCC stage								
I	Reference	–	Reference	–	Reference	–	Reference	–
II	2.81 (2.42–3.26)	<0.001	1.15 (0.93–1.42)	0.193	2.96 (2.53–3.47)	<0.001	1.17 (0.94–1.46)	0.153
III	7.95 (6.85–9.23)	<0.001	2.23 (1.75–2.84)	<0.001	8.57 (7.33–10.03)	<0.001	2.33 (1.81–2.99)	<0.001
Tumor size								
≤2 cm	Reference	–	Reference	–	Reference	–	Reference	–
>2 cm,≤5 cm	2.31 (2.08–2.57)	<0.001	1.40 (1.23–1.60)	<0.001	2.40 (2.16–2.68)	<0.001	1.43 (1.25–1.64)	<0.001
>5 cm	4.81 (4.26–5.43)	<0.001	1.70 (1.45–2.00)	<0.001	4.95 (4.37–5.62)	<0.001	1.70 (1.44–2.00)	<0.001
LN status								
Negative	3.27 (2.97–3.61)	–	Reference	–	Reference	–	Reference	–
Positive	3.47 (2.59–4.65)	<0.001	2.08 (1.83–2.37)	<0.001	3.40 (3.07–3.76)	<0.001	2.12 (1.85–2.41)	<0.001
HRs^‡^								
ER+/PR+	Reference	–	Reference	–	Reference	–	Reference	–
ER+/PR−	1.58 (1.37–1.82)	<0.001	1.31 (1.14–1.51)	<0.001	1.62 (1.40–1.87)	<0.001	1.34 (1.16–1.55)	<0.001
ER−/PR+	1.93 (1.49–2.50)	<0.001	1.98 (1.53–2.58)	<0.001	1.97 (1.51–2.57)	<0.001	2.02 (1.54–2.64)	<0.001
ER−/PR−	2.06 (1.88–2.26)	<0.001	1.77 (1.60–1.95)	<0.001	2.10 (1.91–2.31)	<0.001	1.80 (1.62–1.99)	<0.001
Surgery								
MAS	Reference	–	Reference	–	Reference	–	Reference	–
BCT	0.48 (0.44–0.54)	<0.001	0.76 (0.67–0.86)	<0.001	0.48 (0.43–0.54)	<0.001	0.76 (0.67–0.86)	<0.001
PMBR	0.56 (0.50–0.62)	<0.001	0.71 (0.64–0.79)	<0.001	0.57 (0.51–0.63)	<0.001	0.73 (0.66–0.82)	<0.001
Radiation								
No	Reference	–	Reference	–	Reference	–	Reference	–
Yes	1.30 (1.19–1.42)	<0.001	0.95 (0.85–1.05)	0.287	1.33 (1.21–1.45)	<0.001	0.96 (0.86–1.07)	0.465
Chemotherapy								
No/Unknown	Reference	–	Reference	–	Reference	–	Reference	–
Yes	2.40 (2.06–2.79)	<0.001	1.18 (1.00–1.38)	0.049	2.48 (2.12–2.91)	<0.001	1.18 (1.00–1.40)	0.047

^*^With adjustment for age, race, marital status, year of diagnosis, AJCC stage, tumor size, lymph node status, histological grade, hormone receptor status, surgery, radiation and chemotherapy.

OS, overall survival; BCSS, breast cancer-specific survival; HR^†^, hazard ratios; CI, confidential interval; LN, lymph node; HRs^‡^, hormone receptor status; ER, estrogen receptor; PR, progesterone receptor; MAS, mastectomy; BCT, breast-conserving therapy; PMBR, post-mastectomy breast reconstruction.

## Discussion

By investigating the survival outcomes of young women with operable breast cancer treated with mastectomy, BCT or PMBR in a population of 20,885 patients from the SEER database, our study found that BCT or PMBR had improved OS and BCSS compared with mastectomy for young women with operable breast cancer, which remained significant in subgroup of unmarried patients. In subgroup of married patients, PMBR still conferred better OS and BCSS than mastectomy, but BCT did not. In addition, BCT and PMBR had equal OS and BCSS for young breast cancer patients, which were not affected by marital status.

Previous randomized controlled trials and large retrospective studies have demonstrated that BCT had better or at least equivalent survival outcomes compared with mastectomy (2–5). However, only a low percentage of younger patients has been included and adequately evaluated in these researches. Young breast cancer patients who were considered to have more aggressive tumors and higher risk of local recurrence after breast surgery, the surgical management of breast cancer might be more aggressive even without clear demonstration of benefit (12). Both the European Society of Breast Cancer Specialists (EUSOMA) working group and the fourth international consensus conference for breast cancer in young women recommended breast-conserving surgery as the first option whenever suitable, as it provides same overall survival compared with mastectomy (7, 13). A systemic meta-analysis also declared that BCT provided equivalent survival compared with mastectomy in operable breast cancer patients younger than 40 years old (12). In 2020, Wang et al. (14) noticed that BCT did not have survival benefit compared with mastectomy for young patients with breast cancer; however, the number of young patients in the study was relatively small and the results were only adjusted for tumor size, hormone receptor, HER2 and lymph nodes statuses. As for breast cancer patients who are not suitable for breast conservation, PMBR has been proved to have better or at least equivalent impact on both overall survival and breast cancer recurrence rates compared with mastectomy alone (15). Furthermore, Bezuhly et al. (6) found that immediate breast reconstruction after mastectomy was associated with higher BCSS compared with mastectomy alone among younger women, consistent with the result in our study.

In our finding, there was no significant difference in survival between BCT and PMBR, though PMBR usually brought more injuries to local tissues and needed more time to recover. This result might be attributable to the fact that both BCT and PMBR maintained patients’ body image to some extents and improved their psychosocial life, as the breast is a significant aspect of women’s body image and has an effect in how women are perceived by others or the society as well as in women’s self-perception (16–18). It has been reported that patients with greater psychological stress and less psychosocial support were more likely to have tumor progression and immune dysfunction (9), which might partly explain the result that young breast cancer patients who chose BCT or PMBR had better survival outcomes. After both univariate and multivariate analysis using Cox regression model, we found that marriage was a protective factor to prognosis for young breast cancer patients, which was consistent to the results of previous studies (9, 10). The possible underlying reasons why married patients with breast cancer had better prognosis included greater financial resources, more prompt treatments and more psychological support (10). It has also been documented that patients who are married display less depression and anxiety than those who are unmarried after diagnosis of breast cancer, since a partner can share the emotional burden and provide appropriate social support (19, 20). Therefore, clinical doctors are supposed to pay more attention to assessing and relieving the psychological stress of unmarried young patients with breast cancer as well as maximizing their treatment adherence.

To explore the differences of demographic and pathological factors between married and unmarried young patients, we found that unmarried patients had higher AJCC stage and larger tumor size, but had higher percentage of BCT (see **Supplementary Material 1**). The result that unmarried patients had higher tumor stage and larger tumor size was similar to the findings of other studies (9) and could easily explained, since unmarried patients generally had lesser financial resources or psychological support, impeding them to undergo timely physical examination, obtain better insurance coverage and receive more treatments (21). Although unmarried patients were found to have larger tumor size at diagnosis, the percentage of them who underwent BCT was higher than that of married patients, reflecting the importance of breast conservation for unmarried young patients. To further investigate the effect of marital status in survival outcomes of patients who underwent different surgical approaches, we compared the OS and BCSS of patients in the three surgical groups stratified by marital status. As shown in **Figure 3**, we have noticed that unmarried patients who underwent mastectomy had worst OS and BCSS while married patients who underwent BCT had best OS and BCSS, which can be well explained by the finding that both of being unmarried and undergoing mastectomy are adverse predictors for prognosis of breast cancer patients. Furthermore, BCT conferred survival benefit compared with mastectomy in unmarried young patients but not in the married after eliminating confounding bias *via* multivariate analysis. This result is consistent to our assumption, since unmarried young patients were considered to be more concerned about maintaining the shape of their breast after surgery compared with the married. For patients with breast cancer, young women usually have stronger willing to conserve their breast compared with the older, so as to keep their body image and improve confidence in their psychosocial life. Meanwhile, compared with the married, it is more difficult for unmarried young patients to take a hit when told to dissect their breast, as they often need to face with more psychosocial stress while obtain less psychological support than the married. Therefore, even though unmarried young patients are more likely to have higher tumor stage at diagnosis, breast-conserving surgery should be recommended as the first option whenever suitable.

Unlike studies from single institution which had referral bias unavoidably, our study used SEER database, a large population-based cancer registry containing information from all levels of healthcare institutions, to present a more generalizable environment of clinical practice. There are several limitations in our study. Firstly, as a retrospective study including a large population from SEER database, there might existed data-entry errors and selection bias. Secondly, some information about marital status and prognosis of breast cancer patients could not be accessible in SEER database, including levels of hormone, reproductive history and subsequent treatments. Therefore, we could not further investigate the mechanism of the relationship between marital status and the prognosis of breast cancer patients; however, it might have little influence on the results of our research, which mainly focused on the impact of surgical approaches in survival outcomes for young women with operable breast cancer in different marital statuses. Thirdly, the information of ER and PR status was gathered from various pathology laboratories, possibly increasing bias of the data. Finally, information related to local recurrence and regional recurrence were unavailable in the SEER database, thus we failed to recognize patients with breast cancer recurrence who might have more advanced therapies.

## Conclusion

By investigating the impact of surgical approaches in survival outcomes for young women with operable breast cancer in different marital statuses using the SEER database, our study demonstrated that both BCT and PMBR had improved survival compared with mastectomy for young women with operable breast cancer. The superiority of BCT in survival benefit to mastectomy was seen in unmarried patients but not in married patients. Meanwhile, BCT and PMBR had equal survival benefit for young breast cancer patients, which was not affected by marital status. According to our study, BCT should be recommended as the first option for young women with operable breast cancer whenever suitable; otherwise, PMBR is suggested to maintain the patients’ body image as much as possible.

## Data Availability Statement

Publicly available datasets were analyzed in this study. The data can be found at Surveillance, Epidemiology, and End Results (SEER) database (https://seer.cancer.gov/).

## Ethics Statement

This study was exempted by the ethics committee of Guangdong Provincial People's Hospital because our data were from the SEER database, which is de-identified and open to the public. And the SEER program approved the use of these data without the need for individual subject consent.

## Author Contributions

JZ, CY and YZ were involved in the design and coordination of the study as well as in data analysis, interpretation of results, and drafting the manuscript. KW was in charge of all study procedures. The others participated in the study procedures and critically revised the content of the manuscript. All authors contributed to the article and approved the submitted version.

## Funding

This study is supported by grants from National Natural Science Foundation of China (Grant No. 81871513), Science and Technology Planning Project of Guangzhou City (202002030236), Science and Technology Special Fund of Guangdong Provincial People’s Hospital (No. Y012018218), CSCO-Hengrui Cancer Research Fund (Y-HR2016-067), Guangdong Provincial Department of Education Characteristic Innovation Project (2015KTSCX080), The Fundamental Research Funds for the Central Universities (2020ZYGXZR017) and Guangdong Basic and Applied Basic Research Foundation (2020A1515010346).

## Conflict of Interest

The authors declare that the research was conducted in the absence of any commercial or financial relationships that could be construed as a potential conflict of interest.
