# Supplementary material for: Effects of Surgery on Prognosis of Young Women With Operable Breast Cancer in Different Marital Statuses: A Population-Based Cohort Study
Source: Front Oncol. 2021 Jun 23;11:666316. doi: 10.3389/fonc.2021.666316 (PMC8261040; doi:10.3389/fonc.2021.666316)
Supplement: Supplementary file 1 [file Table_1.docx]

**Supplementary material 1** Comparison of baseline characteristics between married and unmarried patients.

| Characteristics | Patients, No. (%) |  | P value |
| --- | --- | --- | --- |
|  | Married  N=13402 | Unmarried  N=7483 |  |
| Grade  I  II  III/IV  AJCC stage  I  II  III | 998(7.4)  4706(35.1)  7698(57.4)  4055(30.3)  6591(49.2)  2756(20.6) | 562(7.5)  2539(33.9)  4382(58.6)  2060(27.5)  3835(51.2)  1588(21.2) | 0.222  <0.001 |
| Tumor size  ≤2cm  >2cm,≤5cm  >5cm  LN status  Negative  Positive  Surgery  MAS  BCT  PMBR  Radiation  No  Yes  Chemotherapy  No/Unknown  Yes  HRs  ER+/PR+  ER+/PR-  ER-/PR+  ER-/PR- | 5922(44.2)  5901(44.0)  1527(11.4)  6804(50.8)  6404(47.8)  4761(35.5)  3607(26.9)  5034(37.6)  5991(44.7)  7411(55.3)  2368(17.7)  11034(82.3)  7956(59.4)  1368(10.2)  277(2.1)  3801(28.4) | 2900(38.8)  3555(47.5)  992(13.3)  3995(53.4)  3369(45.0)  2657(35.5)  2359(31.5)  2467(33.0)  3219(43.0)  4264(57.0)  1403(18.7)  6080(81.3)  4406(58.9)  794(10.6)  130(1.7)  2153(28.8) | <0.001  0.001  <0.001  0.019  0.052  0.272 |

Abbreviations: MAS, mastectomy; BCT, breast-conserving therapy; PMBR, post-mastectomy breast reconstruction; AJCC, American Joint Committee on Cancer; HRs, hormone receptor status; LN, lymph node.
